# The role of Nd^3+^ concentration in the modulation of the thermometric performance of Stokes/anti-Stokes luminescence thermometer in NaYF_4_:Nd^3+^

**DOI:** 10.1038/s41598-022-27339-9

**Published:** 2023-01-10

**Authors:** K. Maciejewska, L. Marciniak

**Affiliations:** Institute of Low Temperature and Structure Research, Polish Academy of Sciences, Okólna 2, 50-422 Wroclaw, Poland

**Keywords:** Nanoscience and technology, Chemical engineering, Inorganic chemistry

## Abstract

The growing popularity of luminescence thermometry observed in recent years is related to the high application potential of this technique. However, in order to use such materials in a real application, it is necessary to have a thorough understanding of the processes responsible for thermal changes in the shape of the emission spectrum of luminophores. In this work, we explain how the concentration of Nd^3+^ dopant ions affects the change in the thermometric parameters of a thermometer based on the ratio of Stokes (^4^F_3/2_ → ^4^I_9/2_) to anti-Stokes (^4^F_7/2_,^4^S_3/2_ → ^4^I_9/2_) emission intensities in NaYF_4_:Nd^3+^. It is shown that the spectral broadening of the ^4^I_9/2_ → ^4^F_5/2_, ^2^H_9/2_ absorption band observed for higher dopant ion concentrations enables the modulation of the relative sensitivity, usable temperature range, and uncertainty of temperature determination of such a luminescent thermometer.

## Introduction

Extensive studies on luminescent nanoparticles for their use as functional materials, that exhibit an optical response to specific changes in physical or chemical parameters like temperature, pressure, or pH is the major demand of current nanotechnology^1^. This results, among others, from the extraordinary potential of such materials for the diagnosis and treatment of various illnesses, especially cancer diseases^2–6^. Hopes are particularly high for materials that allow remote temperature sensing^7–13^. Exploiting the thermally induced changes in spectroscopic properties of the phosphor luminescence thermometry enables the measurement of temperature at the tissue and even cellular level due to the sub-micro spatial resolution^14–16^. Due to their high physical stability, especially extensively explored for this purpose are the inorganic materials doped with lanthanide ions^4, 17–20^. Ladder-like energy level diagrams of lanthanide ions together with the relatively long lifetime of their metastable excited states offer the possibility of inducing their Stokes and anti-Stokes emission. As it is well known the reduction of the population of the metastable states of lanthanides at elevated temperatures leads to the quenching of their Stokes emission^21^. On the other hand, the thermalization of the higher lying excited states according to the Boltzmann distribution leads to the opposite thermal monotonicity of the emission intensity of the anti-Stokes emission^13^. When the same metastable energy state is emitting state for Stokes emission and the starting point from which thermalization to the higher energy states occurs at elevated temperatures it is possible to achieve the opposite thermal monotonicity of two luminescence signals from one type of dopant ions. Hence the difference in the thermal dependence of those two signals enables the development of the ratiometric luminescence thermometer of high thermal sensitivity. Efficient thermalization with preserving the intense emission intensity from the upper state impose the selection of lanthanide ion of the energy separation between two subsequent energy state lower than 2000 cm^−1^^13^. One of the most commonly used lanthanide ion in luminescence thermometry, due to is unique energy level diagram, is neodymium (Nd^3+^)^22–28^. As proved recently by Suo et al. the unique energy level configuration of Nd^3+^ facilitates the development of Stokes/anti-Stokes ratiometric luminescent thermometers^29–33^. A high concentration of the Nd^3+^ ions facilitates the thermalization process due to the more efficient absorption of the incident radiation and the more efficient light-to-heat conversion related to the heating of the particle. The light-to-heat conversion process in Nd^3+^ doped nanoparticles enables to develop a light-induced nanoheaters^17, 34–37^. Since the energy difference between metastable ^4^F_3/2_ state and upper laying ^4^F_5/2_, ^2^H_9/2_ and then ^4^F_7/2_, ^2^S_3/2_ are relatively low ~ 1000 cm^−1^ the thermalization process itself according to the Boltzmann distribution is more probable to be involved than the energy transfer up-conversion.

Therefore in this work, the influence of Nd^3+^ dopant concentration on the luminescence thermometer exploiting Stokes to anti-Stokes emission intensity ratio will be systematically investigated. In order to minimize the effect of the nonradiative processes associated with the host material, the NaYF_4_ nanoparticles were used in these studies, that is well known for their low phonon energies. The influence of the Nd^3+^ concentration on the thermometric performance of the ratiometric thermometer including relative sensitivity and usable temperature range is analyzed.

## Materials and methods

### Materials preparation

The materials were synthesized by the solvothermal method in oleic acid as solvent. Neodymium(III) oxide (99.999%), yttrium(III) oxide (99.999%) were purchased from Alfa Aesar, sodium fluoride (99.99%), chloric acid (99%), pure oleic acid were purchased from Sigma Aldrich. Sodium hydroxide (99.8%), ethanol (96% pure p.a.), *n*-hexane and chloroform were purchased from POCH S.A. (Poland). All of the chemical reagents were used as received without further purification.

In a 50-mL autoclave, 0.6 g of NaOH was dissolved into 5 mL of deionized water under stirring. Thereafter, an aqueous solution of rare earth chlorides (0.2 mmol) was added. Then, 10 mL of ethanol and 10 mL of oleic acid were added under vigorous stirring. After stirring at 50 °C for 1 h 0.2 mmol aqueous solution of sodium fluoride was added immediately. Finally, 10 mL of ethanol was added into the autoclave after stirring for another 30 min, and the autoclave was sealed and heated at 180 °C for 8 h. The solution was cooled to room temperature and the nanoparticles were collected by centrifugation and washed three times with hexane/ethanol solution. The final product was redispersed in 5 mL of chloroform or for spectroscopic measurements was prepared by drying precipitates at room temperature.

### Methods

Powder diffraction data were obtained using a PANalytical X'Pert Pro diffractometer equipped with an Anton Paar TCU 1000 N Temperature Control Unit using Ni-filtered Cu Kα radiation (*V* = 40 kV and *I* = 30 mA)^38, 39^. Transmission electron microscope (TEM) images were recorded with a Philips CM-20 SuperTwin transmission electron microscope, operating at 160 kV. A drop of the suspension was put on a copper microscope grid covered with carbon. Before the measurement, the sample was dried and purified in a H_2_/O_2_ plasma cleaner for 1 min. The excitation spectra and luminescence decay profiles were obtained using an FLS1000 Fluorescence Spectrometer from Edinburgh Instruments equipped with a 450 W xenon lamp and μFlash lamp as an excitation sources and R5509-72 photomultiplier tube from Hamamatsu in a nitrogen-flow cooled housing as a detector. To carry out the temperature measurement, the temperature of the sample was controlled using a THMS 600 heating–cooling stage from Linkam (0.1 °C temperature stability and 0.1 °C set point resolution). The emission spectra were recorded using 808 nm excitation lines from laser diode (LD of 1.1 W/cm^2^ excitation density) and a Silver-Nova Super Range TEC Spectrometer from Stellarnet (1 nm spectral resolution) as a detector.

## Results and discussion

**Figure 1:**
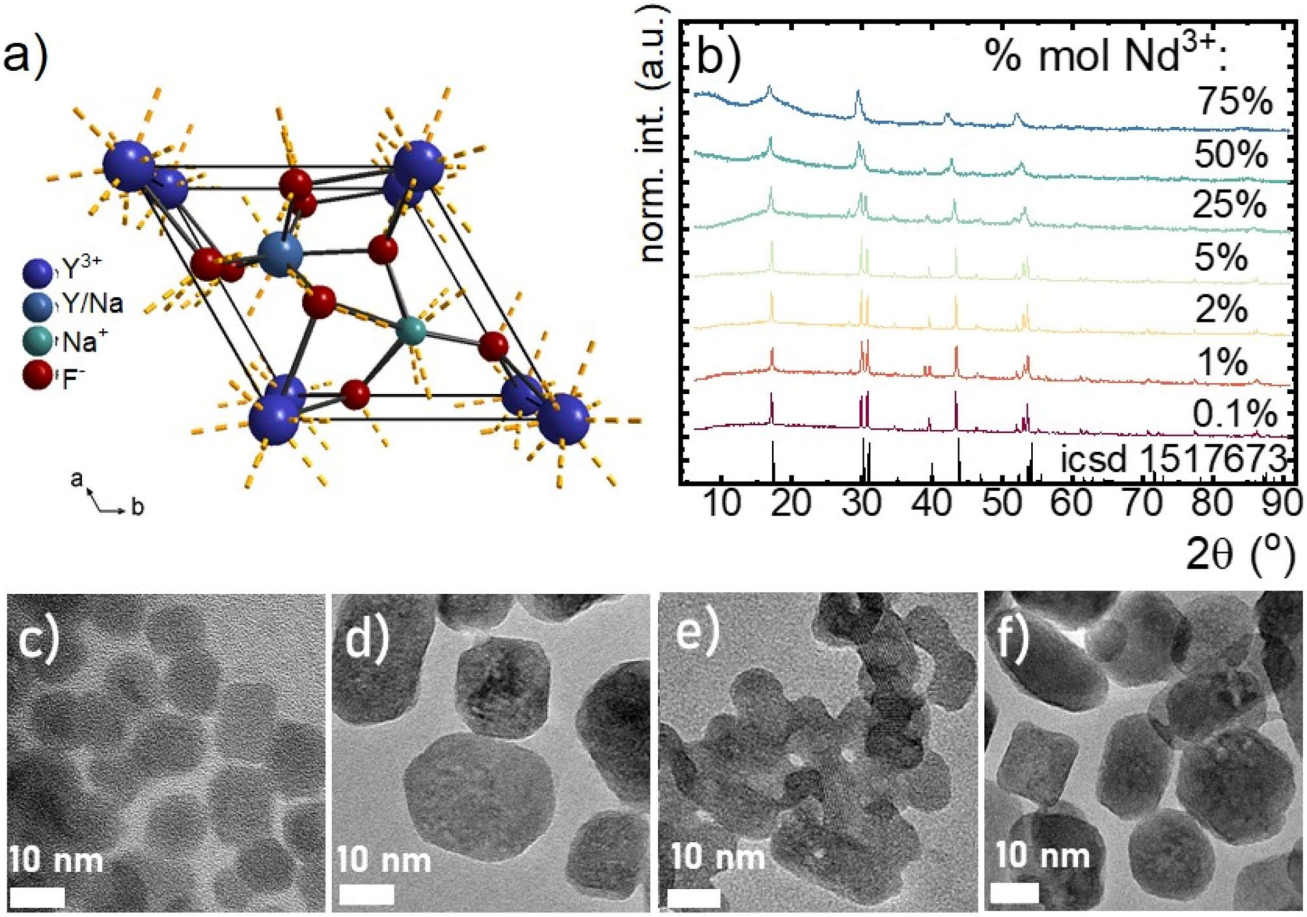
The visualization of the NaYF_4_ crystal unit cell (**a**); X-ray diffraction patterns of NaYF_4_ doped with different concentration of Nd^3+^ (**b**); the representative TEM images of NaYF_4_ nanoparticles doped with 1% (**c**), 5% (**d**), 25% (**e**), 75% (**f**) of Nd^3+^-ions.

In order to analyze the influence of Nd^3+^ dopant concentration on the thermometric properties of this type of luminescence thermometer a series of nanoparticles with different concentrations of Nd^3+^ changes in the range of 0.1–75% in respect to the Y^3+^ ions. As a result of the proposed simple procedure, β-NaY_x_Nd_1−x_F_4_ nanoparticles crystallizing in the hexagonal phase were synthesized. The determination of the symmetry group of these compounds crystallizing in the β-phase is still debatable due to the several possibilities (*P6*, *P6*_*3*_*/m* or *P 2 m*) of assigning the symmetry group^40^. In the case of *P6* (Fig. 1a) crystal structure the RE^3+^ ions (RE^3+^-rare earths) accommodate the first crystallographic position with the nine-fold coordination. The same coordination number is achieved by RE^3+^/Na^+^ ions (in the ratio 3:1) for the *P6*_*3*_*/m* crystallographic position, whereas Na^+^ ions occupy the third sixfold coordinated position. In turn, for the *P6*_*3*_*/m* group, the positions occupied by the rare-earth metal ion (RE^3+^ and RE^3+^/Na^+^) are symmetrically correlated and mixed with each other. In this study, the β-NaYF_4_ of the *P6* space group was used as a reference pattern. As it can be seen in Fig. 1b all of the diffraction reflections correspond to the reference data, and there are no additional peaks that could indicate the presence of another phase or by-products. However, some differences can be observed due to the change in the intensity of the diffraction peaks. An increase in the Nd^3+^ concentration results in a broadening of the reflections that suggests a reduction of the particle size (Fig. 1c). However, TEM images reveal that the size of the materials oscillated in the range of over 15 nm, 23 nm, 15 nm to 20 nm for NaYF_4_:1%Nd^3+^, NaYF_4_:5%Nd^3+^, NaYF_4_:25%Nd^3+^ to NaYF_4_:75%Nd^3+^, respectively (Fig. 1c–f, see also Supplementary Fig. S1 for particles size distribution). TEM image analysis confirmed the preparation of crystalline nanoparticles with a narrow grain distribution and a non-aggregated form. Only for nanoparticles with a dopant ion concentration of 25% Nd^3+^ subtle of the aggregation was observed.

**Figure 2:**
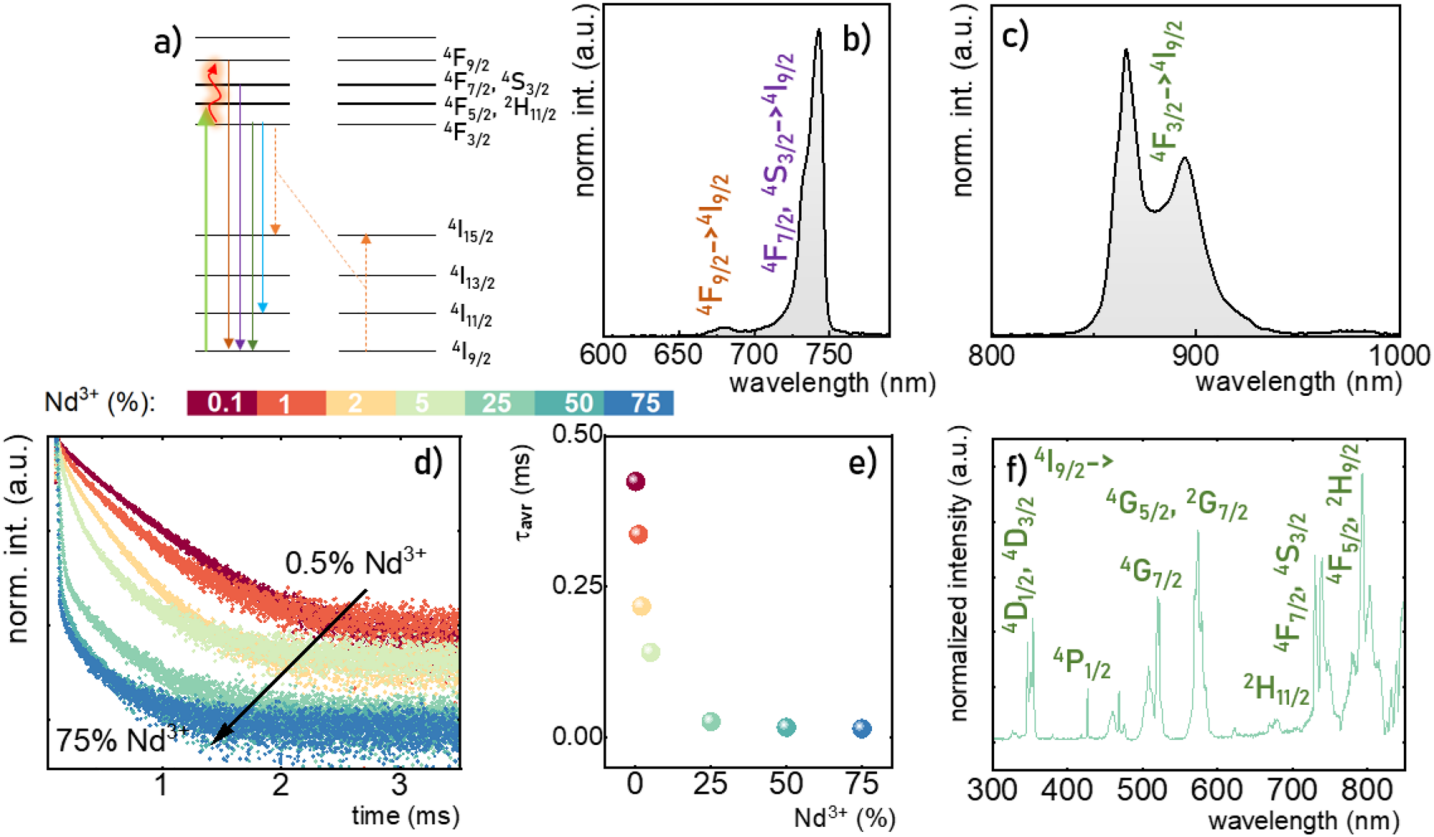
Simplified energy diagram of Nd^3+^ ions (**a**); the representative Stokes (**b**) and anti-Stokes (**c**) emission spectra upon 808 nm excitation of the NaYF_4_:1%Nd^3+^ nanoparticles; the luminescence decay profiles of the ^4^F_3/2_ state (measured for ^4^F_3/2_ → ^4^I_11/2_ transition at 123 K) (**d**); the τ_avr_ monitored at 123 K as a function of Nd^3+^ concentration (**e**); and the representative excitation spectrum of NaYF_4_:1%Nd^3+^ nanoparticles measured at 123 K (**f**).

Despite the small difference in the particle size was observed when the Nd^3+^ concentration was changed NaYF_4_:x%Nd^3+^ (x = 0.1, 1, 2, 5, 25, 50, 75) this effect should not affect significantly the spectroscopic properties of the analyzed nanoparticles, as shown recently by Trejgis et al.^41^. Therefore the change in their luminescence properties can be discussed in terms of the Nd^3+^ concentration effect. To understand this effect the simplified energy level diagram of Nd^3+^ ions is presented in Fig. 2a. Upon 808 nm excitation, the electrons from the ground ^4^I_9/2_ to the ^4^F_5/2_, ^2^H_9/2_ state are transferred, followed by the nonradiative depopulation to the metastable ^4^F_3/2_ state. The radiative relaxation of this state to the ^4^I_9/2_, ^4^I_11/2_, ^4^I_13/2_ energy levels results in the occurrence of emissions bands centered around 890 nm, 1060 nm, and 1325 nm, respectively. An increase in the temperature results in the thermalization of the upper ^4^F_5/2_, ^2^H_9/2_ and ^4^F_7/2_, ^2^S_3/2_ and ^4^F_9/2_ states. This process enables the generation of the ^4^F_5/2_, ^2^H_9/2_ → ^4^I_9/2_, ^4^F_7/2_, ^2^S_3/2_ → ^4^I_9/2_ and ^4^F_9/2_ → ^4^I_9/2_ electronic transitions corresponding to the emission bands at 800 nm, 740 nm and 690 nm, respectively. A shortening of the average distance between Nd^3+^ ions associated with the increase in dopant concentration increases the probability of the {^4^F_3/2_, ^4^I_9/2_} ↔ {^4^I_15/2_, ^4^I_15/2_} cross relaxation that leads to the quenching of the emission intensity and shortening of the lifetime of the ^4^F_3/2_ state^42–46^. Both anti-Stokes and Stokes emission bands can be observed simultaneously (Fig. 2b,c). However, the anti-Stokes luminescence is less intense. Therefore to analyze the shape of the emission spectra both parts of the spectrum were presented separately. Although the spectral position of the emission band is independent of dopant concentration the shape of the ^4^F_3/2_ → ^4^I_9/2_ band changes significantly (see also Supplementary Figs. S2 and S3). At elevated Nd^3+^ concentration the intensity of the emission lines corresponding to the R_1_ and R_2_ Stark levels of the ^4^F_3/2_ state to the Z_5_ Stark component of the ^4^I_9/2_ level decreases due to the energy reabsorption (Supplementary Fig. S4). The metastable ^4^F_3/2_ state plays a crucial role in the generation of both Stokes (radiative depopulation of ^4^F_3/2_ state) and anti-Stokes (as a platform for thermalization of higher laying states) luminescence of Nd^3+^ ions. Therefore it is important to analyze the influence of the Nd^3+^ ions concentration on the lifetime of the ^4^F_3/2_ state. As shown in Fig. 2d the exponential luminescence decay profile can be found for low dopant concentration and an increase in Nd^3+^ amount results in a deviation from exponential shape due to the cross relaxation process. Therefore to perform a qualitative analysis the average lifetime was calculated as follows:

1 $$\tau_{avr} = \frac{{A_{1} \tau_{1}^{2} + A_{2} \tau_{2}^{2} }}{{A_{1} \tau_{1} + A_{2} \tau_{2} }},$$

where A_1_, A_2_, τ_1_ and τ_2_ are the parameters determined from the fitting of the decay profiles with bi-exponential functions:

2 $$I\left( t \right) = I_{0} + A_{1} e^{{ - \frac{t}{{\tau_{1} }}}} + A_{2} e^{{ - \frac{t}{{\tau_{2} }}}} ,$$

here I_0_ represents the initial emission intensity. For the nanoparticles doped with 0.1% Nd^3+^ ions the longest τ_avr_ = 0.430 ms was observed, which shortens with Nd^3+^ to τ_avr_ = 0.336 ms, 0.217 ms, 0.141 ms, 0.026 ms, 0.017 ms and 0.014 ms for 1%, 2%, 5%, 25%, 50% and 75% of Nd^3+^, respectively (Fig. 2e). The lack of change in the number of components in the excitation spectra of Nd^3+^ ions in NaYF_4_:Nd^3+^ for the ^4^I_9/2_ → ^2^P_1/2_ electronic transition proves that Nd^3+^ ions consequently occupy only one crystallographic position (Y^3+^ site) in NaYF_4_ structure (Fig. 2f, see also Supplementary Fig. S5). Deeper insight into the change of the local crystallographic surrounding of the Nd^3+^ ions with an increase of dopant concentration can be provided by the analysis of the intensities ratio of ^4^I_9/2_ → ^4^G_5,7/2_ band (hypersensitive band) to the ^4^I_9/2_ → ^4^D_1/2_ bands. In the NaYF_4_:Nd^3+^ nanoparticles the ratio changes from 0.13 for 0.5% Nd^3+^ up to 0.40 for 75% Nd^3+^ confirming the decrease in local symmetry and an increase in covalency associated with the enlargement of the dopant amount (Supplementary Fig. S6).

**Figure 3:**
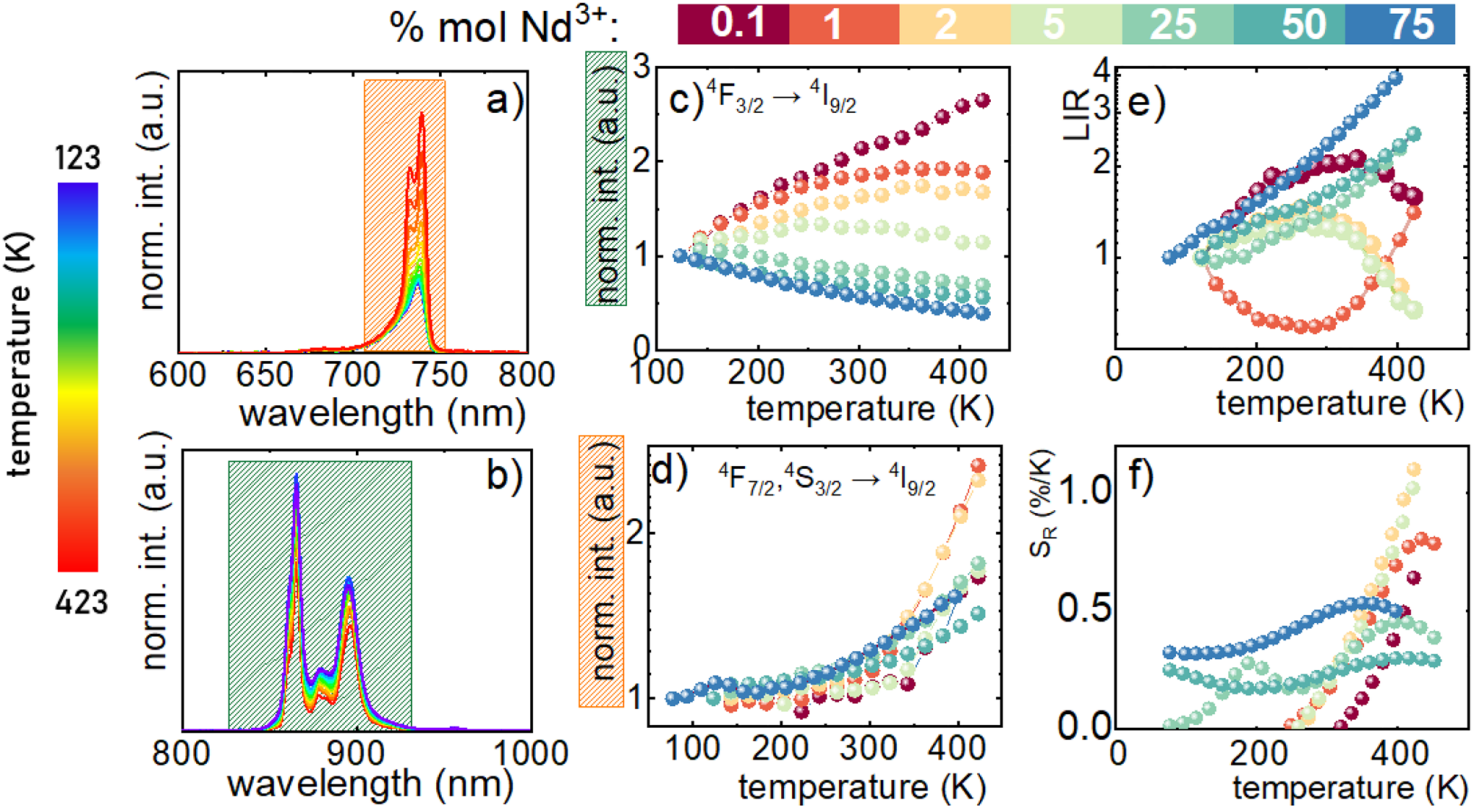
The anti-Stokes (**a**); and Stokes (**b**) parts of emission of NaYF_4_:1%Nd^3+^ nanoparticles upon 808 nm excitation; the impact of the concentration of Nd^3+^ ions on thermal evolution of normalized integral emission intensities of ^4^F_3/2_ → ^4^I_9/2_ (**c**) and ^4^F_7/2_,^4^S_3/2_ → ^4^I_9/2_ (**d**) emission bands of NaYF_4_: Nd^3+^ nanoparticles; the temperature dependent LIR values of NaYF_4_:Nd^3+^ nanoparticles (**e**); the corresponding S_R_ (**f**).

To understand how the temperature changes affect the spectroscopic properties of the NaYF_4_:Nd^3+^ nanoparticles their emission spectra in both anti-Stokes (Fig. 3a) and Stokes (Fig. 3b) part of spectra were analyzed as a function of temperature in the range of 83–423 K (Supplementary Fig. S7). The representative spectra presented in Fig. 3a reveal that the intensity of the ^4^F_7/2_,^4^S_3/2_ → ^4^I_9/2_ band increases gradually at elevated temperatures which can be understood since ^4^F_7/2_,^4^S_3/2_ state is thermally coupled with ^4^F_3/2_ and its population increases with temperature. On the other hand, the emission intensity of the ^4^F_3/2_ → ^4^I_9/2_ emission band decreases as a consequence of the reduction of the ^4^F_3/2_ state population via two effects: (i) its nonradiative depopulation and (ii) thermalization of the upper laying ^4^F_5/2_, ^2^H_9/2_ state (followed by thermalization of the ^4^F_7/2_,^4^S_3/2_ state). The analysis of the thermal dependence of the integrated emission intensity of this band for different concentrations of dopant ions reveals that it is strongly affected by the Nd^3+^ amount (Fig. 3c). In the case of low Nd^3+^ concentration an increase in temperature results in almost threefold enhancement of the integrated intensity of this band. However when the concentration increases the rate of thermal enhancement gradually reduces up to NaYF_4_:5%Nd^3+^ for which only a bare change in emission intensity was observed. For higher Nd^3+^ amounts the opposite thermal dependence was found and the strongest thermal quenching of ^4^F_3/2_ → ^4^I_9/2_ emission intensity was found for NaYF_4_:75%Nd^3+^. As stated above the Stokes emission of Nd^3+^ is expected to be quenched at elevated temperature. The thermal enhancement of the intensity of this band is a consequence of the excitation wavelength used (λ_exc_ = 808 nm). Although, this is commonly used optical excitation for Nd^3+^ doped phosphors in the case NaYF_4_:Nd^3+^ the maximum of the ^4^I_9/2_ → ^4^F_5/2_, ^2^H_9/2_ absorption band is slightly shifted toward blue with the maxima around 796 nm (Supplementary Fig. S2). Hence the λ_exc_ = 808 nm reached the sideband of this band. An increase in temperature results in a broadening of the absorption band and thus more efficient absorption of the incident light, resulting in an increase in emission intensity (Supplementary Fig. S8). When the concentration of the Nd^3+^ increases the spectral broadening of this absorption band can be found (Supplementary Fig. S9) and excitation wavelength is efficiently absorbed already at low temperature. Thus the thermal broadening of the absorption band does not affect so strongly the thermal dependence of integral emission intensity. It is worth noticing that the optimization of dopant concentration enables the counteraction of these two processes and achieves almost thermally stable ^4^F_3/2_ → ^4^I_9/2_ luminescence of Nd^3+^ ions in NaYF_4_:Nd^3+^ nanoparticles. In the case of the ^4^F_7/2_,^4^S_3/2_ → ^4^I_9/2_ luminescence less spectacular dopant effects were observed (Fig. 3d). Independently of dopant concentration and enhancement in the emission intensity was found. However, for a higher Nd^3+^ amount, the enhancement was slightly lower due to the previously described depopulation of the ^4^F_3/2_ state. The difference in the thermal change of the anti-Stokes and Stokes part of the spectrum enables the development of the ratiometric luminescence thermometer in which the luminescence intensities ratio (LIR) is considered as a thermometric parameter:

3 $$LIR = \frac{{\int_{{720\,{\text{nm}}}}^{{740\,{\text{nm}}}} {Nd^{3 + } } \{^{4} F_{7/2} ,^{4} S_{3/2} \to^{4} I_{9/2} \} d\lambda }}{{\int_{{870\,{\text{nm}}}}^{{900\,{\text{nm}}}} {Nd^{3 + } } \{^{4} F_{3/2} \to^{4} I_{9/2} \} d\lambda }}.$$

Due to the meaningful impact of dopant concentration on the thermal dependence of ^4^F_3/2_ → ^4^I_9/2_ and ^4^F_7/2_,^4^S_3/2_ → ^4^I_9/2_ emission bands the LIR is strongly affected by the Nd^3+^ amount (Fig. 3e). Only above 25% Nd^3+^ the monotonic thermal dependence of LIR in the whole analyzed temperature range can be found, whereas for lower dopant amount increase of LIR is followed by its reduction at elevated temperature. Only in the case of the NaYF_4_:1%Nd^3+^ the opposite thermal dependence was found. The change in the thermal monotonicity of the thermometric parameters reduces the temperature range in which a given thermometer can be applied. This is due to the fact that a reliable temperature readout can be provided when the given value of the parameter LIR can be unequivocally assigned to a given temperature. The quantification of the observed thermal changes in LIR can be performed by the relative sensitivity calculation using the following equation:

4 $$S_{R} = \frac{1}{LIR}\frac{\Delta LIR}{{\Delta T}}100\% ,$$

where ΔLIR represents the change of LIR corresponding change of temperature by ΔT. The S_R_ was calculated in the temperature range in which an increase of LIR was observed (Fig. 3f). As can be seen the higher values of the NaYF_4_:2%Nd^3+^ reaching around S_R_ = 1.1%/K at 410 K. For a higher dopant amount the reduction of the maximal S_R_ was observed. However, it should be noticed here that in the case of the nanoparticles with a high Nd^3+^ amount (> 5%) the S_R_ reached higher values at temperatures below 250 K comparing to a low dopant counterpart. The repeatability of the LIR readout within a several heating–cooling cycles was also confirms high accuracy of temperature readout (Supplementary Fig. S10). It should be also mentioned that the particle size may affect the thermometric properties of luminescence thermometers. However, as shown in the previously published studies^38^, the dopant concentration plays a far more important role than particle size. The clear correlation between the dopant concentration and the thermometric parameters of NaYF_4_:Nd^3+^ is a clear confirmation of this hypothesis.

**Figure 4:**
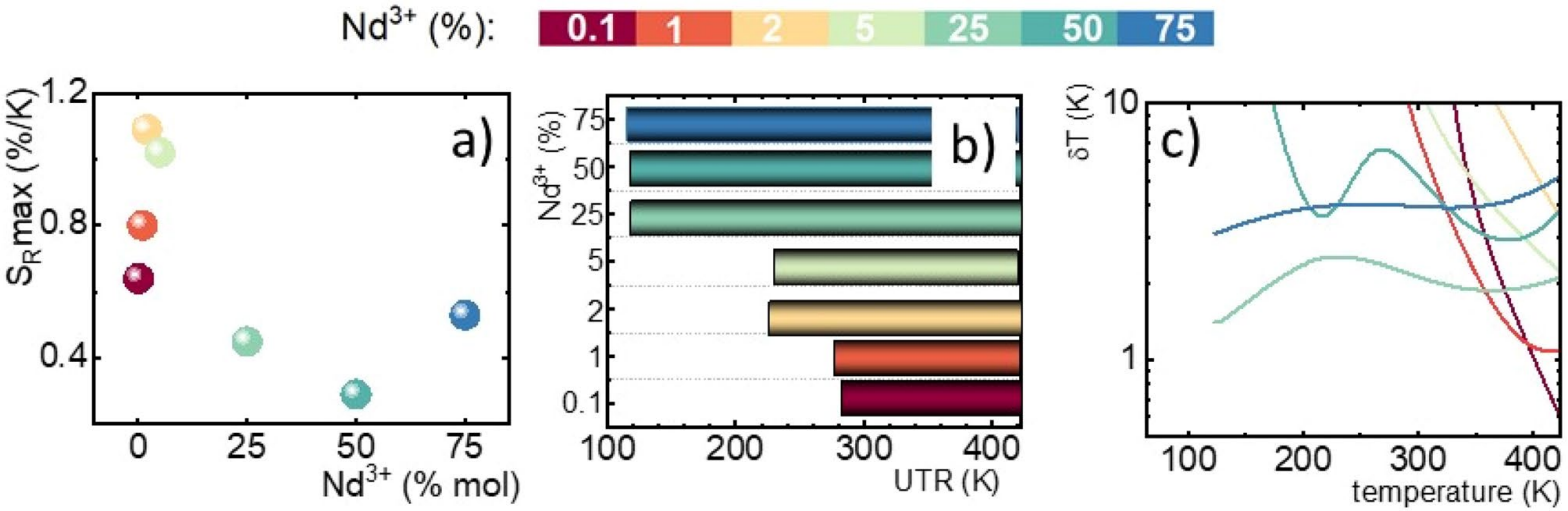
The maximum relative sensitivity (**a**); an usable temperature range (UTR) (**b**); and temperature determination uncertainty (**c**) for different Nd^3+^ concentration in NaYF_4_:Nd^3+^ nanoparticles.

Depending on the requirement of the particular application different thermometric parameters should be considered. To facilitate this the maximal S_R_, usable temperature range (UTR) and the temperature determination uncertainty (δT) of the luminescence thermometer based on anti-Stokes to Stokes LIR in NaYF_4_:Nd^3+^ with different concentrations of Nd^3+^ ions were analyzed (Fig. 4). The S_R_ max increases monotonically with dopant concentration up to NaYF_4_:2%Nd^3+^ followed by the gradual reduction of its value (Fig. 4a). Above 25%Nd^3+^ the S_R_ remains almost independent of dopant concentration at around S_R_ = 0.4%/K. Actually, this value of the S_R_ is relatively high comparing the ratiometric thermometer based on R_1_ and R_2_ emission lines of Nd^3+^ (S_R_ ~ 0.1–0.2%/K)^17, 27, 47, 48^, however, lower than the SR for the LIR of the ^4^F_7/2_,^4^S_3/2_ → ^4^I_9/2_ to ^4^F_5/2_, ^2^H_9/2_ → ^4^I_9/2_ emission bands^29–33, 49, 50^. It is evident that although the S_R_ decreases with dopant concentration the usable temperature range simultaneously is extended. While the NaYF_4_:0.1%Nd^3+^ can be applied only in the 290–423 K temperature range the increase of Nd^3+^ above 25% results enables to extend of the UTR to 123–423 K. Therefore depending on the requirement of application these two parameters should be appropriately balanced. It should be noted that although some of the luminescent thermometers reveal high relative sensitivity their low emission intensity results in a low signal-to-noise ratio and thus in the high uncertainty of LIR determination (δLIR/LIR). The calculations of temperature determination uncertainty (δT) performed for NaYF_4_:Nd^3+^ as follows:

4 $$\delta T=\frac{ 1}{{S}_{R}}\frac{\delta LIR}{\text{LIR}}100\%,$$

reveal that the lowest δT ~ 2 K in the whole analyzed temperature range was found for NaYF_4_:25%Nd^3+^. Although for a higher concentration of Nd^3+^ very similar S_R_ was achieved the reduced emission intensity, especially of the Stokes emission significantly affects the δT.

## Conclusions

In this work, the development of a ratiometric luminescence thermometer based on the intensities ratio of Stokes to anti-Stokes emission in NaYF_4_:Nd^3+^ was described. For this purpose, the effect of Nd^3+^ ion concentration on the temperature variation of ^4^F_3/2_ → ^4^I_9/2_ and ^4^F_7/2_, ^4^S_3/2_ → ^4^I_9/2_ band luminescence intensity was investigated. As shown due to the spectrally narrow ^4^I_9/2_ → ^4^F_5/2_, ^2^H_9/2_ absorption band, an increase in ^4^F_3/2_ → ^4^I_9/2_ emission intensity was observed for low concentrations of dopant ions when using the commercially used 808 nm excitation. An increase in the concentration of Nd^3+^ ions and the associated broadening of the absorption band caused compensation for this effect, and above 5%Nd^3+^ the intensity of this band decreased with increasing temperature. On the other hand, the temperature dependence of the ^4^F_7/2_,^4^S_3/2_ → ^4^I_9/2_ band reveals only slight dopant effect indicating that this emission generation process is mainly single-ion in nature and related to the thermalization of the ^4^F_/2_,^4^S_3/2_ level from the ^4^F_5/2_,^2^H_9/2_ and ^4^F_3/2_ levels. As a result, the highest relative sensitivity of S_R_ = 1.1%/K was recorded for NaYF_4_:0.1%Nd^3+^. Above 25%Nd^3+^ S_R_ remains almost independent of dopant concentration S_R_ ~ 0.4%/K. However, in contrast to what was observed for low concentrations of Nd^3+^, for high concentrations of Nd^3+^, the LIR increased monotonically over the full range of temperatures analyzed, significantly widening the useful temperature range over which such a thermometer can be used. In summary, luminescence thermometers based on LIR of Stokes to anti-Stokes emission in NaYF_4_:Nd^3+^ nanoparticles are characterized by attractive thermometric properties whose thermometric performance can be modulated by the concentration of Nd^3+^ ions.

## Supplementary Information

Supplementary Information.

## Data Availability

The datasets used and/or analysed during the current study available from the corresponding author on reasonable request.
