# Supplementary material for: The role of Nd3+ concentration in the modulation of the thermometric performance of Stokes/anti-Stokes luminescence thermometer in NaYF4:Nd3+
Source: Sci Rep. 2023 Jan 10;13:472. doi: 10.1038/s41598-022-27339-9 (PMC9832010; doi:10.1038/s41598-022-27339-9)
Supplement: Supplementary file 1 — Supplementary Information. [file 41598_2022_27339_MOESM1_ESM.docx]

**Supporting Information**

**The role of Nd^3+^ concentration in the modulation of the thermometric performance of Stokes /anti-Stokes luminescence thermometer in NaYF_4_:Nd^3+^**

K. Maciejewska^1*^, L. Marciniak^1*^

^1^Institute of Low Temperature and Structure Research, Polish Academy of Sciences, Okólna 2, 50-422 Wroclaw, Poland

* corresponding author: [*k.maciejewska@intibs.pl*](mailto:k.maciejewska@intibs.pl)

[*l.marciniak@intibs.pl*](mailto:l.marciniak@intibs.pl)

KEYWORDS: luminescent nanoparticles, luminescent nanothermometry, Nd^3+^ based LT, anti-Stokes emission, Stokes emission


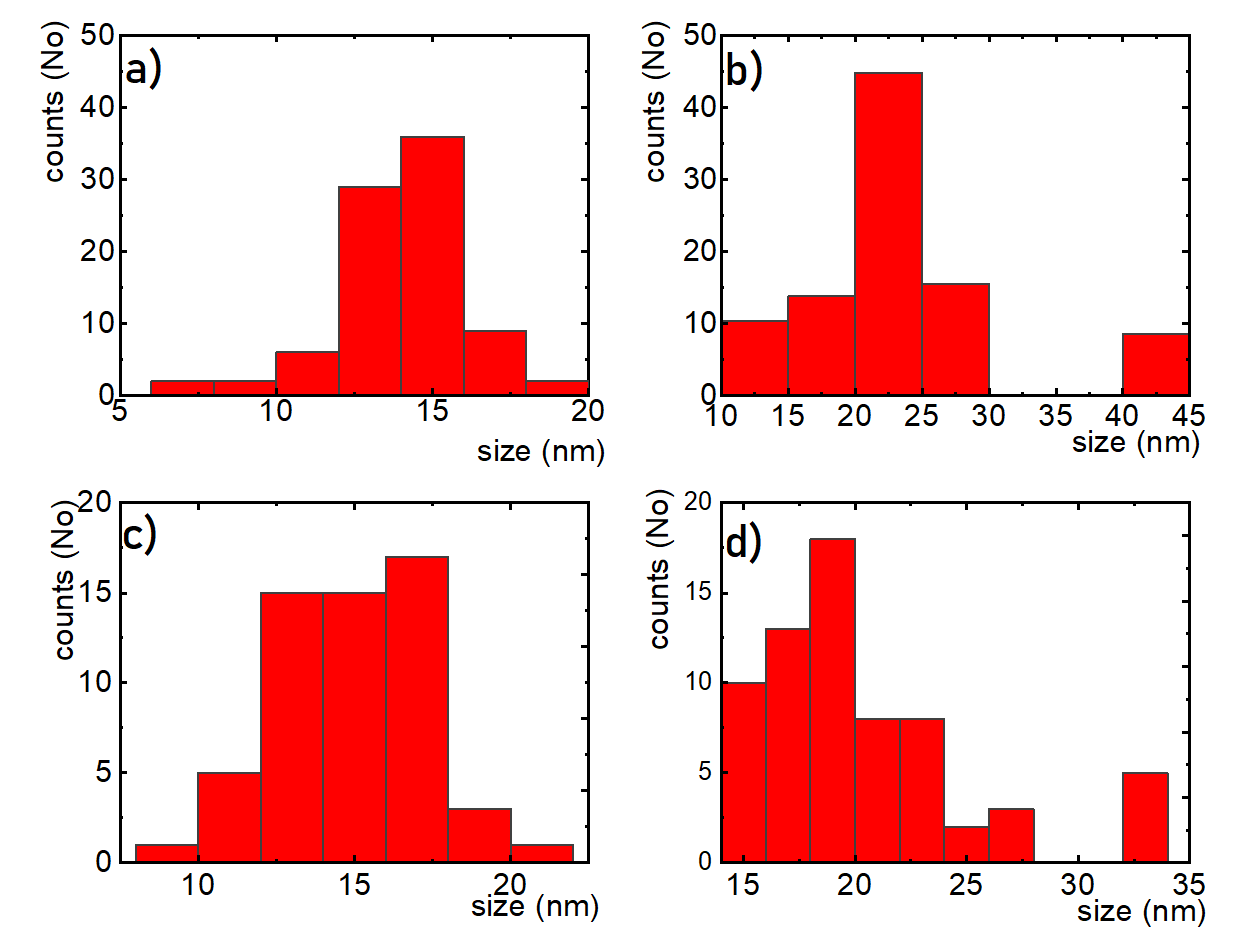


# Figure S1. Particle size distribution histograms of NaYF_4_:1%Nd^3+^ (a), NaYF_4_:5%Nd^3+^ (b), NaYF_4_:25%Nd^3+^ (c) and NaYF_4_:75%Nd^3+^ (d) nanoparticles determined from TEM images.

#
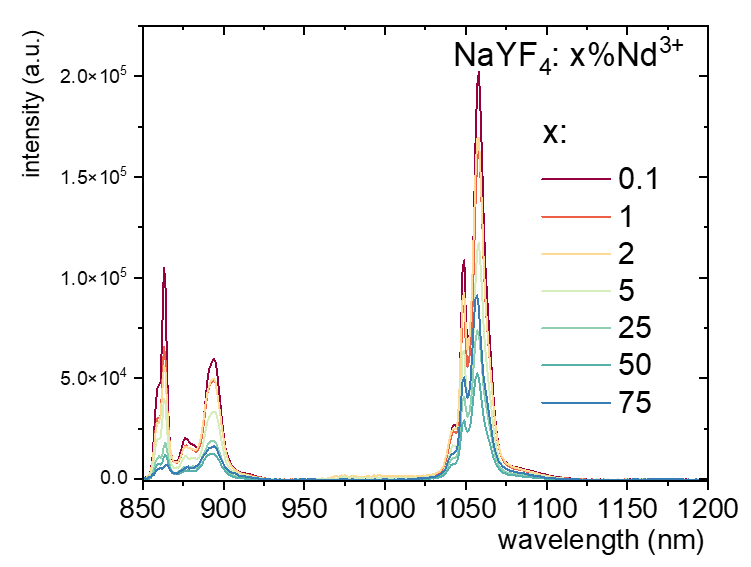


# Figure S2. Comparison of emission spectra of NaYF_4_:Nd^3+^ measured upon 808 nm excitation at 123 K.


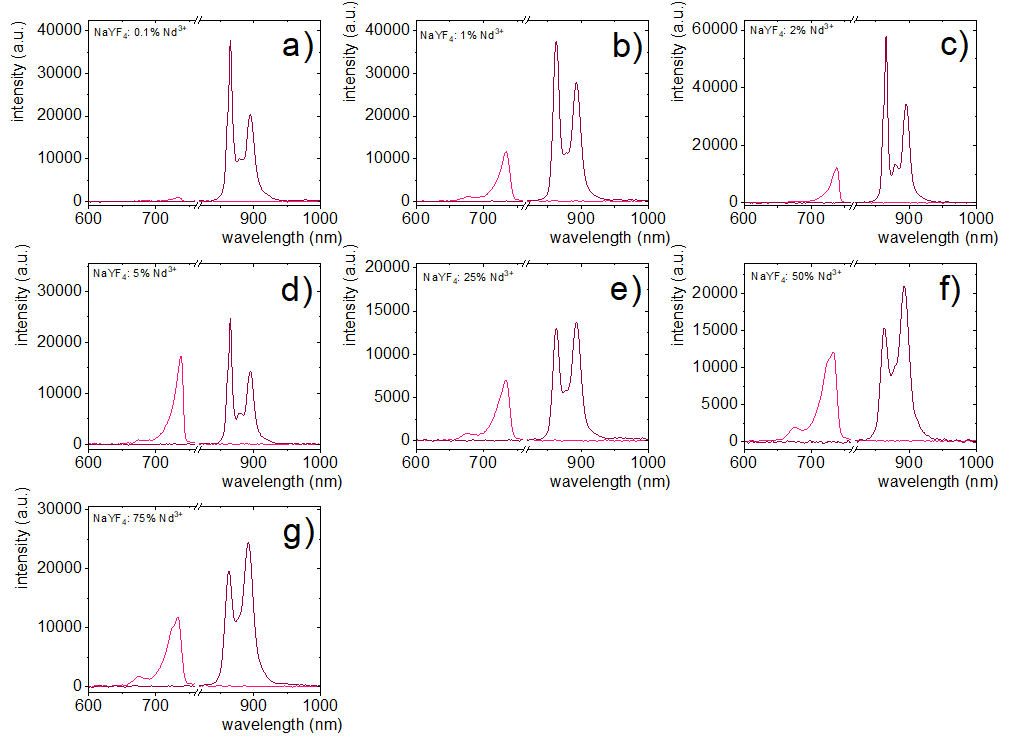


**Figure S3.** Comparison of Stokes and anti-Stokes part of the emission spectra of NaYF_4_:Nd^3+^ measured at 123 K upon the same experimental conditions for: a)-0.1%Nd^3+^; b)-1%Nd^3+^; c)-2%Nd^3+^; d)-5%Nd^3+^; e)-25%Nd^3+^; f)-50%Nd^3+^; g)-75%Nd^3+^


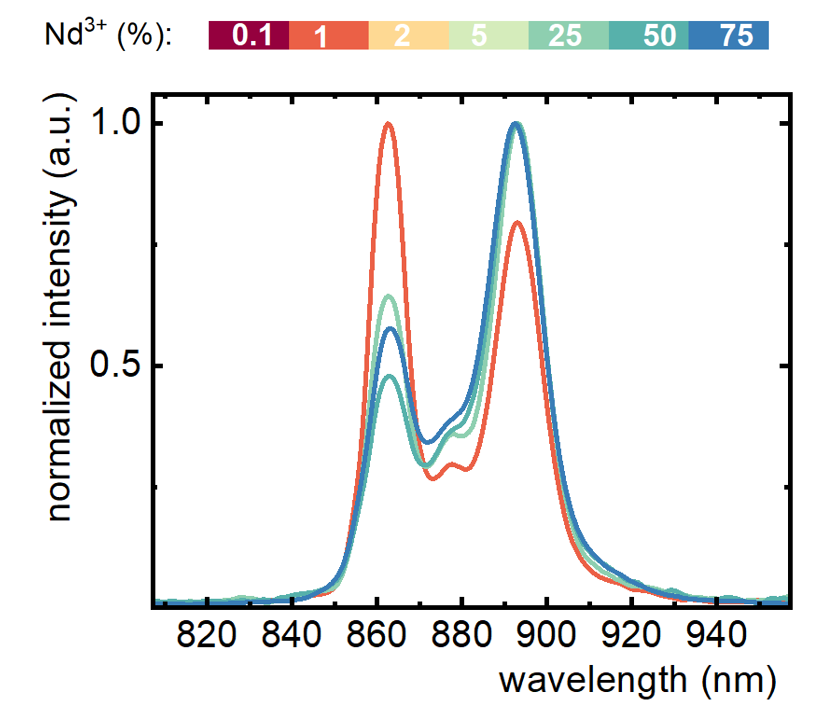


**Figure S4**. The comparison of the shape of the ^4^F_3/2_→^4^I_9/2_ emission band of Nd^3+^ ions in NaYF_4_:Nd^3+^ for different concentration of dopant ions.


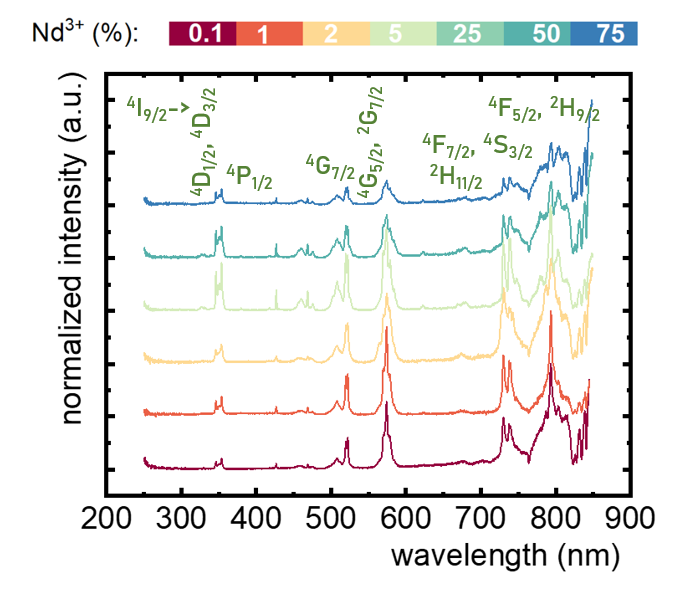


**Figure S5**. The comparison of the excitation spectra of NaYF_4_:Nd^3+^ for different concentration of Nd^3+^ ions


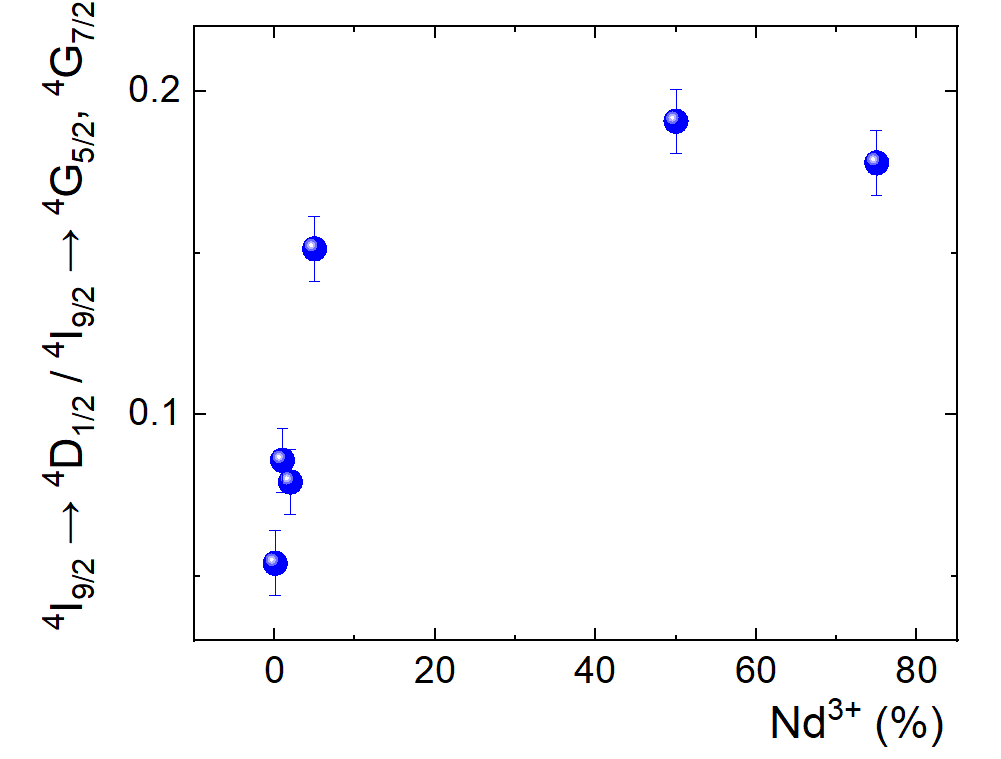


**Figure S6**. The influence of Nd^3+^ concentration on the ^4^I_9/2_ → ^4^G_5,7/2_ to the ^4^I_9/2_ → ^4^D_1/2_ absorption intensities ratio in NaYF_4_:Nd^3+^ nanoparticles.


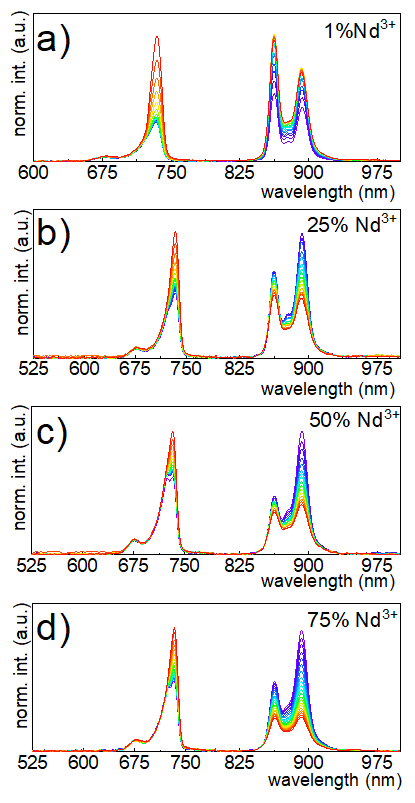


**Figure S7**. The thermal evolution of emission upon 808 nm excitation line of 1% Nd^3+^ (a); 25%Nd^3+^ (b); 50%Nd^3+^ (c) and 75%Nd^3+^ (d) of NaYF_4_ nanoparticles.


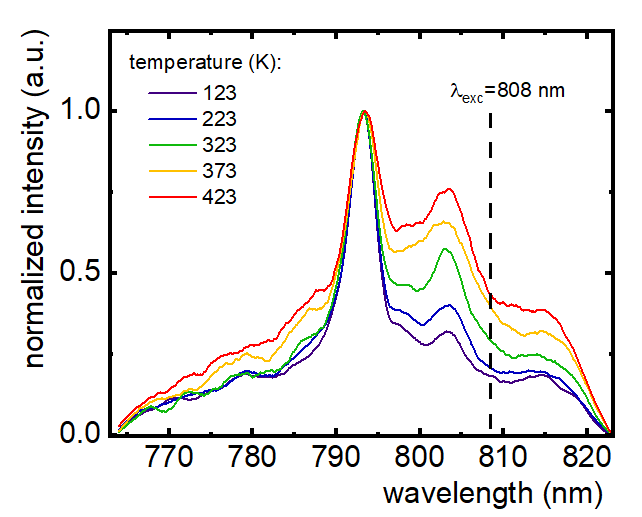


**Figure S8**. The comparison of the shape of the ^4^I_9/2_→^4^F_5/2_, ^4^S_3/2_ excitation band of NaYF_4_:0.1%Nd^3+^ measured at different temperatures (λ_em_=1060 nm).


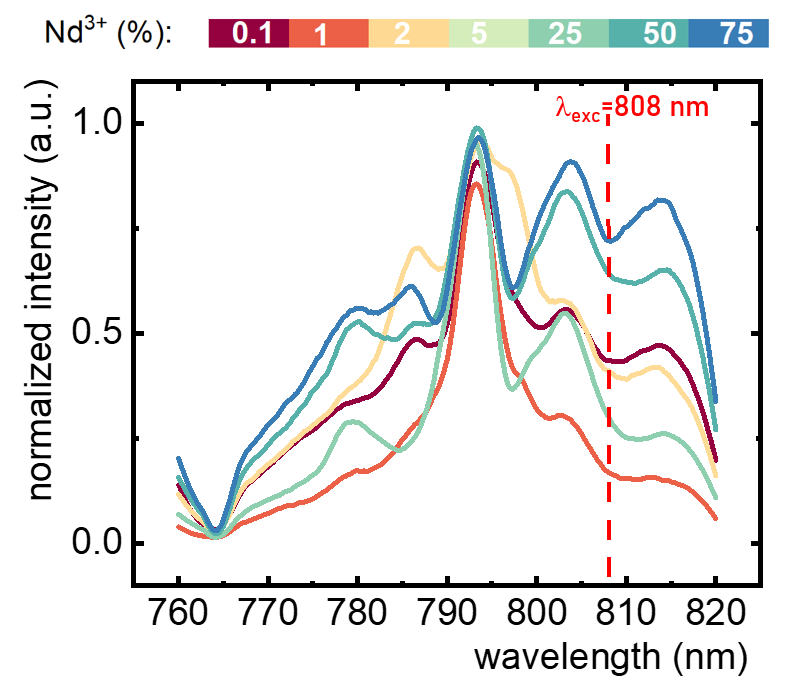


**Figure S9**. The comparison of the shape of the ^4^I_9/2_→^4^F_5/2_, ^4^S_3/2_ excitation band of NaYF_4_:Nd^3+^ for different concentration of Nd^3+^ ions (λ_em_=1060 nm).


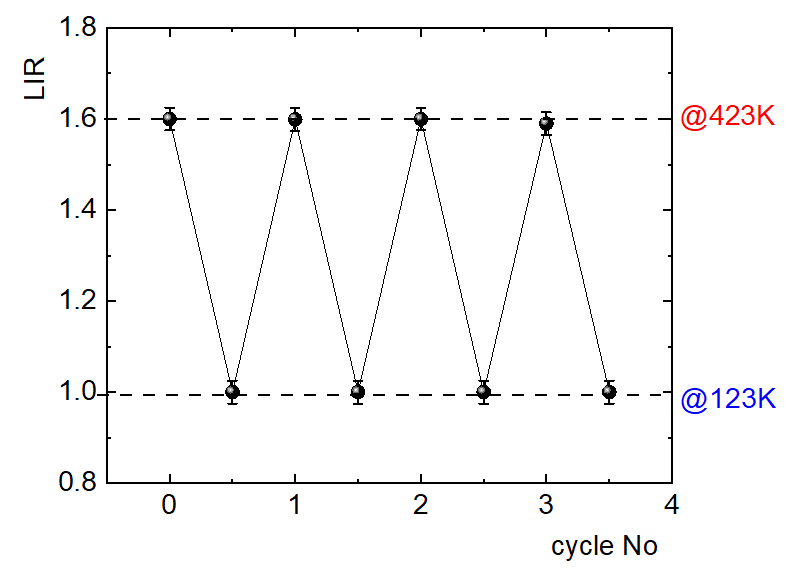


**Figure S10**. LIR calculated within heating-cooling cycles measured for NaYF_4_:1%Nd^3+^ nanocrystals.
